# The Tradeoff between Travel Time from Home to Hospital and Door to Balloon Time in Determining Mortality among STEMI Patients Undergoing PCI

**DOI:** 10.1371/journal.pone.0158336

**Published:** 2016-06-23

**Authors:** Riccardo Di Domenicantonio, Giovanna Cappai, Paolo Sciattella, Valeria Belleudi, Mirko Di Martino, Nera Agabiti, Francesca Mataloni, Roberto Ricci, Carlo Alberto Perucci, Marina Davoli, Danilo Fusco

**Affiliations:** 1 Department of Epidemiology, Lazio Regional Health Service, Roma, Italy; 2 Cardiology Unit, Local health authority Roma E, Roma, Italy; Osaka University Graduate School of Medicine, JAPAN

## Abstract

**Background:**

In ST-segment elevation myocardial infarction (STEMI), even in presence of short door to balloon time (DTBT), timely reperfusion with percutaneous coronary intervention (PCI) is hampered by pre-hospital delays. Travel time (TT) constitutes a relevant part of these delays and may contribute to worse outcomes.

**Objective:**

To evaluate the relationship between TT from home to hospital and DTBT on 30-day mortality after PCI among patients with STEMI.

**Methods:**

We enrolled a cohort of 3,608 STEMI patients with a DTBT within 120 minutes who underwent PCI between years 2009 and 2013 in Lazio Region (Italy). We calculated the minimum travel time from residential address to emergency department where the first medical contact occurred. We defined system delay as the sum of travel time and DTBT time. Logistic regression models, including clinical and demographic characteristics were used to estimate the effect of TT and DTBT on mortality.

**Results:**

Among patients with 0–90 minutes of system delay, TT above the median value is positively associated with mortality (OR = 2.46; P = 0.009). Survival benefit associated with DTBT below the median results only among patients with TT below the median (OR for DTBT below the median = 0.39; P = 0.013), (OR for interaction between TT and DTBT = 2.36; p = 0.076).

**Conclusion:**

TT affects survival after PCI for STEMI, even in the presence of health care systems compliant with current guidelines. Results emphasize the importance of health system initiatives to reduce pre-hospital delay. Utilization of TT can contribute to a better estimate of patient mortality risk in the evaluation of quality of care.

## Introduction

In ST-segment elevation myocardial infarction (STEMI), reperfusion with primary percutaneous coronary intervention (PCI) is a time-sensitive process, as any reperfusion delay is associated with an increase in mortality [1,2]. Time from arrival at hospital to first balloon inflation during PCI, door-to-balloon time (DTBT), predicts survival in patients with STEMI and is a process core measure of hospital’s quality of care [3,4]. Several quality improvement initiatives focus on hospital delays [5–7]. Recently, the role of pre-hospital delay (the time before patient arrival at hospital) on mortality after PCI for STEMI, has received increasing attention [8–10]. Optimal goal for treatment with PCI is defined in current guidelines as within 90 minutes or less from the first medical contact (FMC), including both hospital and pre-hospital delays [11]. Transport time may constitute a relevant part of the pre-hospital delay, and driving travel time (TT) from home to hospital of FMC is a reliable surrogate measure of transport time available from Geographic Information System [12–17].

Assessing the association between TT and mortality after PCI, and better understanding the role of pre-hospital delay and DTBT in determining mortality, can highlight the importance of earlier presentation to hospital and provide a more accurate estimate of patient mortality risk.

We conducted a study to evaluate the effect of TT on 30-day mortality after PCI among patients with STEMI. The first aim was to evaluate the effect of TT among patients treated within a 90 minute system delay (SD—defined as sum of TT and DTBT). The second objective was to evaluate the role of TT on the relationship between DTBT and mortality among patients treated within a 120 minute DTBT.

## Methods

### Study design and data source

We carried out a retrospective cohort study in Lazio Region, Italy, using the regional health information systems. We retrieved data routinely collected from: the Hospital Information System, which includes data from discharge abstracts, demographic, and clinical information for all hospital admissions within Lazio Region; the RAD-Esito Information System (RAD), which includes additional data for AMI patients (i.e. systolic blood pressure and time of procedure); the Emergency Room Information System, which includes additional information on time of admission; and the Mortality Information System, which includes information on date and cause of death, coded according to ICD-9 codes. Vital status was ascertained by integrating all hospital information system using a unique patient anonymous identifier deriving from information on persons’names, date and place of birth and gender, according to Italian privacy legislation. Data are routinely collected by the Health Information System Regional Department of the Lazio Regional Health Service, who anonymize all the records prior to the analysis performed by our Department of Epidemiology of the Lazio Regional Health Service, which is the regional referral center for epidemiological research.

### Cohort definition

**Fig 1:**
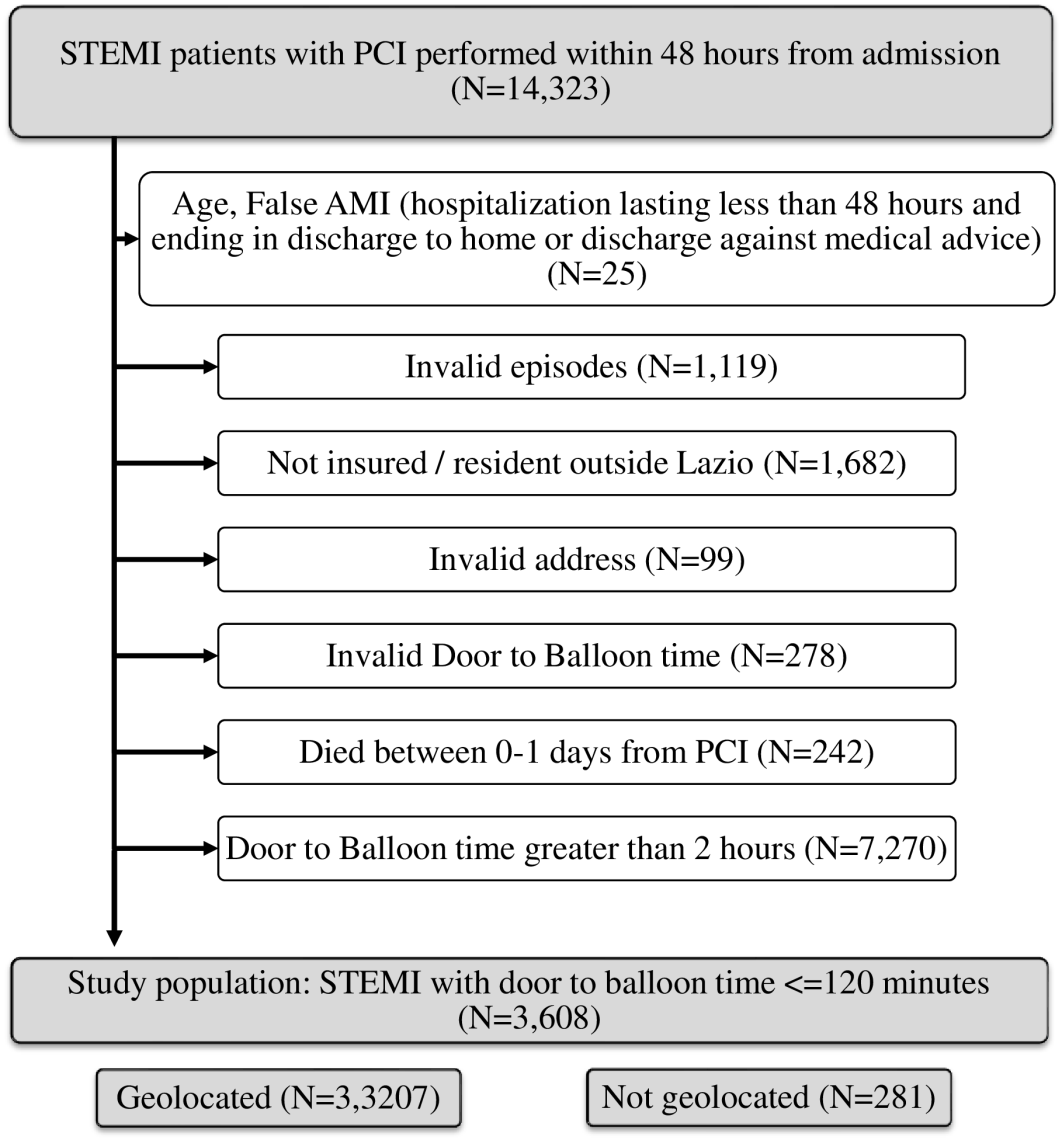
Flow chart for selection of study population. Lazio Region, years 2009–2013. AMI = acute myocardial infarction; PCI = percutaneous coronary intervention; STEMI = ST segment elevation myocardial infarction.

We selected patients aged 35–100 years, discharged with a diagnosis of STEMI (ICD-9-CM: 410.1–410.6, 410.8) between 1 January 2009 and 30 November 2013 [18–19] who underwent PCI (ICD-9-CM: 00.66, 36.01, 36.02, 36.05–36.07) with a DTBT of less than 2 hours. The DTBT time interval and the systolic blood pressure were measured from the patient’s first hospital access. If subsequent STEMI hospitalizations occur within 28 days of the first hospital admission (index admission), only the first admission was included, under the assumption that the following admissions were part of the same STEMI episode. Among 14,323 STEMI patients underwent PCI within 48 hours from admission, about 20% were not insured or not resident in Lazio or were characterized by invalid episodes, 50% had a DTBT greater than two hours. After application of all exclusion criteria, this study included 3,608 patients. Information on travel time was absent for 281 patients (7.8%) due to unsuccessful geocoding of patient addresses. A flow chart showing the procedure to select study population is reported in Fig 1.

### Clinical characteristics and comorbidities

Information about systolic blood pressure at admission was retrieved from RAD. Comorbidities were obtained from the health information systems from both the index and the two years preceding admissions according to a standardized methodology [20]. They include history of previous AMI, cardiovascular risk factor (i.e. diabetes, hypertension, lipid metabolism disturbances) and comorbidities (i.e. chronic obstructive pulmonary disease, other forms of ischemic heart disease, cerebrovascular diseases, cancer). List of conditions and relative ICD-9-CM codes used for identification of comorbidities are reported on S1 Table.

### Travel time from home to hospital of admission

The residential addresses of enrolled patients were considered at the beginning of each year of the study period. The standardization of patient addresses and geocoding were performed using the ESRI ArcGIS software package (ESRI, Redlands, California). Addresses of acute-care general hospitals active during the study period were used to geocode facilities. After geocoding, a road network-based route analysis was conducted to calculate the minimum driving travel time (in minutes) from patient address to hospital. The travel times were calculated based on the speed limits for different roads. We applied a method to take into account the effect of traffic in the area of the municipality of Rome and to estimate a measure of travel time adjusted for traffic. The effect of traffic in the municipality of Rome was estimated among several time bands (8.00–21.59, 22.00–23.59 and 0–7.59) which were assumed as optimal to explain differences in traffic during daytime in the city. Details on methodology to take into account traffic effect are reported in S1 File.

### Exposures definition

Time from first hospital access to first balloon inflation was defined as door-to-balloon time (DTBT). We defined system delay (SD) as the sum of travel time and DTBT. The travel time and DTBT were categorized into two classes according to their median values.

### Study outcome

The main outcome studied was death (all-cause mortality) within 30-days from the date of the PCI.

### Statistical analysis

We performed two main analyses with the cohort data. In the first (sub-cohort), we included only patients with SD ≤ 90 minutes and evaluated the effect of TT. In the second (main cohort), we included all patients with DTBT ≤ 120 minutes and evaluated the effect of TT, DTBT and their interaction.

Logistic regression models were used to estimate the effect of TT and DTBT on mortality, adjusting for several characteristics. The covariates in the models include age class, gender, individual clinical characteristics, presentation (Emergency Medical Service vs direct) and time of the admission day (08.00–21.59 time band vs 22.00–23.59 and 24.00–07.59). A stepwise procedure was used to select clinical characteristics associated with outcomes (p-entry = 0.05, p-stay = 0.1) adjusting for age class and gender, without the inclusion of the exposure of interest. We also checked, among characteristics selected, association with exposures throughout change-in estimate methodology. In this analysis, the variables resulted associated with the outcome, but not with the exposure are excluded to increase parsimony and avoiding over parametrization of the model [21]. In this predictive model, we then included the effect of TT and DTBT as dichotomous variable. To evaluate the presence of interaction we considered a significance level of 0.08 to reject the null hypothesis [22]

To test the effect of hierarchical structure with patients nested within hospitals, we fitted a random intercept logistic regression model. For a variance components test, a Wald test was performed on the statistic obtained by dividing the estimated variance components by their standard error. The corresponding one-sided p-value was evaluated. Two sensitivity analysis was performed on the < = 120 minutes DTBT cohort, the first using the value of 60 minutes as the threshold to dichotomize the DTBT distribution, the second applying a restriction on events occurred during night hours. We calculated and reported the average speed, average length of route and average travel time for all combination of address of origin and destination classified as follows: metropolitan, when origin or destination address is lying in the municipality of Rome, countryside if origin or destination is out of the municipality of Rome. We also calculated the ratio between the average speed for each combination of type of route and time band and the overall average speed. All statistical analyses were carried out using SAS version 9.2 (SAS institute, Cary, NC, USA). We used lincom command available on Stata version 12 (StataCorp. 2011. Stata Statistical Software: Release 12. College Station, TX: StataCorp LP) to test the effect of DTBT among patients with TT above the median throughout linear combination of TT and DTBT coefficients.

## Results

Table 1 reports the characteristics of the sub-cohorts and the main cohort according to mutually exclusive exposure classes. TT and DTBT median values resulted respectively in 13 and 54 minutes for sub-cohort and 14 and 72 minutes for the main cohort. Accounting traffic effects lead to a marked reduction of average speed in metropolitan routes when compared to all routes (22.0 Km/h versus 36.2 Km/h respectively), this effect is more pronounced focusing on the average speed during the time band 8–21.59 (17.3 Km/h) (data reported in the S2 Table). In this time band, accounting for traffic effect, the average speed in the metropolitan area is the half of the regional speed (data reported in the S1 Fig). Patients with higher travel time were characterized by a higher prevalence of males, younger age, lower frequency of systolic pressure under 100 mmHG, higher frequency of presentation by Emergency Medical Services and admission between 8:00 and 21:59. This observation was consistent across both sub-cohort and main cohort.

**Table 1:** Characteristics of the 281 STEMI not geo-located and of the 3,608 STEMI selected according to system delay ≤ 90 minutes (subcohort) or door to balloon time ≤ 120 minutes (main cohort). Lazio Region, years 2009–2013.

	Not geo-located		Sub-cohort (SD < = 90 minutes)						Main cohort (DTBT < = 120 minutes)									
			TT < = median		TT >median		All (°)		TT and DTBT < = median		TT >median		DTBT >median		TT and DTBT >median		All(°)	
**N (%)**																		
	281		985	(48.9)	750	(37.2)	2,016		740	(20.5)	931	(25.8)	952	(26.4)	704	(19.5)	3,608	(°)
**Travel time (Minutes)**																		
Mean (st. dev.)			7.6	(3.9)	24.0	(8.8)	14.7	(10.4)	8.0	(3.9)	27.6	(12.6)	7.4	(3.8)	27.0	(12.9)	17.3	(13.5)
Median			7.0		21.0		13.0		8.0		23.0		7.0		23.0		14.0	
**DTB time (Minutes)**																		
Mean (st. dev.)	68.6	(29.9)	56.4	(19.2)	44.2	(16.7)	53.6	(21.8)	49.2	(16.5)	47.3	(16.9)	95.0	(13.9)	93.5	(13.7)	70.9	(27.9)
Median	71.0		59.0		46.0		54.0		53.0		49.0		95.0		93.0		72.0	
**Gender (%)**																		
Male	80.8		75.0		80.5		77.9		75.8		80.3		74.8		78.3		77.6	
**Age (Years)**																		
Mean (st. dev.)	61.8	(11.3)	64.4	(12.0)	62.3	(12.2)	63.3	(12.0)	64.3	(11.8)	62.4	(12.3)	64.7	(12.4)	63.0	(12.4)	63.5	(12.2)
**Age class (%)**																		
35–64	62.3		50.2		57.7		54.7		50.4		58.3		50.4		58.4		54.9	
65–84	34.2		45.6		39.2		41.6		45.5		38.0		44.1		37.4		40.7	
85+	3.6		4.3		3.1		3.7		4.1		3.7		5.5		4.3		4.3	
**SBP class (%)**																		
< = 100 mmHg	10.3		14.1		12.9		13.1		14.2		12.0		13.2		10.1		12.3	
**Presentation (%)**																		
EMS	56.6		53.5		61.1		56.8		54.3		59.6		46.2		47.3		52.4	
**Time of the admission day (%)**																		
08.00–21.59	78.3		64.0		92.1		76.4		69.5		92.3		54.3		87.4		75.5	
22.00–23.59	8.2		8.5		1.5		5.9		8.1		1.7		10.1		2.8		6.0	
24.00–07.59	13.5		27.5		6.4		17.7		22.4		6.0		35.6		9.8		18.5	
**Cardiovascular risk factor (%)**																		
Diabetes	3.6		3.8		3.9		3.8		3.5		4.1		4.1		3.3		3.8	
Diabetes *	14.6		15.6		14.3		15.0		15.7		15.2		18.6		14.9		16.1	
**Comorbidities (%)**																		
Other forms of IHD heart disease	5.3		3.6		3.1		3.6		3.8		3.3		2.7		3.3		3.4	
Cerebrovascular diseases	2.1		2.6		1.9		2.3		2.3		2.2		3.3		2.8		2.6	
Cancer	2.9		4.4		3.3		3.8		4.3		3.2		3.5		3.7		3.6	
**30-day mortality (%)**																		
	2.5		2.0		3.3		2.6		1.4		2.9		3.5		3.1		2.7	

SBP = Systolic blood pressure EMS = Emergency Medical Service IHD = Ischemic heart disease TT = Travel time DTBT = Door to balloon time SD = System delay

° Percentages do not sum to 100% due to missing values

* Index admission.

Table 2 reports the results of logistic regression analysis of the sub-cohort of patients with SD lower or equal to 90 minutes. Travel time above the median is positively associated with 30–day mortality in crude (OR = 1.66; p = 0.094) and adjusted model (OR = 2.46; P = 0.009), although, for the former the p-value exceeds the significance level of 0.05.

**Table 2:** Logistic regression analysis for < = 90 minutes system delay sub-cohort. Crude and adjusted 30-day mortality odds ratio.

Model	Parameter	Category	Odds ratio estimate	95% Confidence limits		P
Crude (univariate)						
	Travel time	> 14 minutes	1.66	0.92	3.02	0.0940
Adjusted						
	Travel time	> 14 minutes	2.46	1.25	4.86	0.0093
	Gender	Male	0.68	0.35	1.31	0.2454
	Age class	65–84 years vs 35–64 years	4.81	2.05	11.3	0.0003
		> 84 years vs 35–64 years	19.62	6.42	59.9	< .0001
	Systolic blood pressure	≤100 mmHg	4.21	2.19	8.10	< .0001
	Presentation	E.M.S vs. Direct	1.14	0.57	2.27	0.7056
	Hour of admission	22.00–23.59 vs 08.00–21.59	1.80	0.50	6.45	0.3691
		24.00–07.59 vs 08.00–21.59	1.11	0.43	2.85	0.8313
	Comorbidities	Other heart conditions*	7.65	1.76	33.2	0.0066

EMA, Emergency Medical Service;

* Index admission.

Table 3 reports the results of logistic regression analysis on patients with DTBT< = 120 minutes (main cohort). The unadjusted model shows a direct association between DTBT and 30-day mortality (OR for DTBT of 0–72 minutes vs. > 72 minutes = 0.48; P = 0.053). Results of the adjusted model indicate that, among early presenters, DTBT is negatively associated with study outcome, with 30-day mortality odds ratio respectively equal to 0.39 (P = 0.013). Moreover, the effect of short DTBT is different among the strata of travel time (OR for Interaction term = 2.36; p = 0.076) and became null in patients with travel time above the median (OR for linear combination of travel time and interaction term = 0.92; p = 0.797).

**Table 3:** Logistic regression analysis for < = 120 minutes door to balloon time (main cohort). Crude and adjusted 30-day mortality odds ratio.

Model	Parameter	Category	Odds ratio estimate	95% Confidence limits		P
Crude (univariate)						
	Travel time	> 14 minutes	1.19	0.78	1.80	0.4236
	Door to balloon time	0–72 minutes	0.48	0.38	1.01	0.0531
Adjusted						
	Travel time	> 14 minutes	1.15	0.63	2.10	0.662
	Door to balloon time	0–72 minutes	0.39	0.16	0.82	0.013
	Travel time * Door to balloon time		2.36	0.91	6.10	0.076
	Gender	Male	0.71	0.45	1.14	0.154
	Age class	65–84 years vs 35–64 years	3.20	1.83	5.60	< .0001
		> 84 years vs 35–64 years	9.78	4.65	20.55	< .0001
	Systolic blood pressure	≤100 mmHg	3.57	2.22	5.73	< .0001
	Presentation	E.M.S vs. Direct	1.55	0.95	2.52	0.077
	Hour of admission	22.00–23.59 vs 08.00–21.59	1.21	0.45	3.21	0.709
		24.00–07.59 vs 08.00–21.59	1.04	0.56	1.93	0.898
	Comorbidities	Other heart conditions	5.30	1.61	17.43	0.006
		Diabetes*	1.98	1.22	3.21	0.006
		Cerebrovascular diseases	2.56	1.23	5.35	0.012
		Cancer	2.15	0.98	4.73	0.057
Linear combinations of coefficients (Adjusted model)	Door to balloon time * Interaction terms		0.92	0.50	1.68	0.797

EMS, Emergency Medical Service;

* Index admission.

Sensitivity analysis based on a threshold of 60 minutes for DTBT, confirmed the results obtained in the main analysis (OR for DTBT of 0–60 minutes vs. > 60 minutes = 0.40; p = 0.045 and OR for interaction terms equal to 2.40; p = 0.111). Random intercept multilevel regression analysis did not show the presence of a hierarchical structure of data in either model by random intercept multilevel regression analysis (Wald test on between-hospitals variance, p = 0.223 and p = 0.222 for < = 120 minutes DTBT cohort and < = 90 minutes SD sub-cohort, respectively). Results of the sensitivity analysis on events occurred during night hours are reported in supplementary materials (data reported in S3 Table). We found a higher estimates of effect of travel time during 22.00–7.59 time band when compared to 8.00–21.59 time band, with mortality odds ratios for travel time respectively equal to 2.65 (p = 0.08) and 1.37 (p = 0.23).

## Discussion

In the last two decades, the effect of time to treatment on the benefit of PCI after STEMI has been largely investigated by measuring DTBT. To explore an alternative method of evaluation, several studies shifted their focus to system delay, which includes all components of delay modifiable by the health care system, both pre-hospital delay and DTBT [9,10,23]. Current guidelines (American College of Cardiology Foundation/American Heart Association Task Force on Practice Guidelines) promote broad initiatives to improve health system readiness and response to STEMI, focused on the continuum of care from EMS activation to PCI. Health care systems are expected to treat patients with PCI within 90 or 120 minutes from the first medical contact (FMC) for patients who initially arrive at PCI-capable or to a non—PCI-capable hospital, respectively [11]. Travel time from patient residence to hospital was previously used to estimate the proportion of the population with timely access to PCI facilities [12–14], the nearest emergency room (ER), or cardiac and stroke centers [15,16]. Travel time resulted also associated with both out- and in-hospital mortality and long-term mortality for acute myocardial infarction (AMI) [17].

In the absence of delay measures gathered by Emergency Medical Service (EMS), we evaluated the effect of travel time from home to hospital of FMC on 30-day mortality in a cohort of patients undergoing PCI for STEMI. We selected a sub-cohort with system delay (defined as sum of travel time and DTBT) lower or equal to 90 minutes and evaluated the effect of pre-hospital system delay among patients experiencing a system delay compatible with guideline recommendations. Moreover, we estimated the effect of travel time and hospital delay among patients experiencing a hospital system delay of 120 minutes or less. We excluded patients with DTBT greater than 120 minutes since we want to assess the effect of travel time in patients timely treated with PCI, thus, the 120 minutes DTBT threshold, recommended by guidelines, appeared an optimal reference value to select STEMI patients. We believe this approach is optimal to include the patients who had the most to gain with respect to myocardial salvage and maximize the relevance of pre hospital system of care in STEMI patients treated with PCI.

Our findings indicate that travel time, a relevant component of pre-hospital system delay, is positively associated with 30-day mortality for STEMI after PCI. This observation was consistent across the two cohorts we analyzed.

Results from sub-cohorts of patients who underwent PCI within 90 minutes of system delay highlighted a strong effect of travel time on 30-day survival. Those STEMI patients received timely reperfusion because, according to guideline recommendations, PCI were performed within 90 minutes from FMC. This result confirms that pre-hospital delay plays an important role in STEMI patient’s survival, even for those patients who received early PCI in health care systems compliant with current guidelines.

Moreover, among patients with hospital delays lower than or equal to 120 minutes (including between hospital transfer time), we observed that mortality benefit from short hospital delay is limited to those patients with travel time below the median. This result also implies that even in the presence of a reduced DTBT, travel time to the hospital is a relevant factor. Those finding is consistent with results reported by Shiomi et al. who demonstrated that short door to balloon time was independently associated with a lower risk of a composite of death and congestive heart failure in patients with early presentation but not in those with delayed presentation [23].

Both results confirm and emphasize the importance of earlier presentation to hospital and the promotion of health system initiatives that can contribute to reduced pre-hospital delay. The more relevant could be the promotion of ambulance transfer respect to private vehicle transfer and the optimization of process-of-care so that EMS vehicles can or should bypass non-PCI hospitals to reach a PCI hospital directly.

This study presents some limitations. Firstly, travel times adjusted for traffic effects may differs from actual travel times, especially in metropolitan area. Ambulance service data, including travel time and address of origin and destination of the transport would be optimal to validate the methodology we used to adjust for traffic effects, the linkage with such external data will be the aim of further research. Secondly, it is understood that not all patients are at place of residence when STEMI occurs, so we considered the possible misclassification of travel times. A sensitivity analysis for characteristics likely to be associated with staying at home when STEMI occurs (older age, events occurring during the evening, weekend or holidays) would confirm this misclassification. We performed a sensitivity analysis applying restriction on events occurred during night hours on the < = 120 minutes cohort. With such approach, we are confident that the most of the patients experienced the STEMI event at home. Results reported in supplementary materials (Part D Sensitivity Analysis, S3 Table) highlighted that estimates of effect of travel time during night hours is higher when compared to the other time band, supporting the hypothesis that exposure misclassification, is likely to be non-differential. However, we were unable to perform other stratified analysis due to the lack of statistical power (low number of outcomes). Thirdly, we also used travel time for patients transported by EMS, who are likely to be characterized by worse clinical status. Thus, we adjusted for this factor in the analysis and assumed that travel time to the E.D. by private car and EMS are comparable. Thirdly, several clinical information that should be included in the analysis (for example: presence of anterior STEMI, information about the culprit artery, timing of antiplatelets) are not available in our data. Moreover, due to a lack of information on thrombolysis, we cannot ascertain that all PCI procedures we selected are primary PCI. About this point, we retain that this aspect does not bias our analysis because, short hospital delay before PCI suggests that thrombolysis is not likely to have been performed or have failed among the <90 minute system delay cohort. Fourthly, this study includes only who have already survived the pre-hospital phase. Accordingly, high risk patients may have been selectively excluded from study population, leading to results confounded by survival bias [9,23,24]. Descriptive statistics of the cohort suggest that prevalence of risk factor are lower in late presenter compared to early presenter, confirming such picture. Finally, given that the prediction model building was performed using a stepwise procedure, the p-value of 0.05 may not be as adequate as desired given the amounts of tests that are performed simultaneously. This approach could lead to an increased rate of obtaining false positive results. The main strength of the study relies on the fact that, to our knowledge, the effect of travel time in relation to outcome of treatment of a relevant time sensitive condition like STEMI, has not been previously measured. The effect estimate for this novel risk factor comes from a population-based health information system cohort of patients of one entire Region of Italy. Most importantly, to reduce clinical heterogeneity, a relevant problem in studies based on administrative data, we excluded patients with less severe conditions (DTBT greater than two hours).

There is increasing interest in the development and implementation of outcome and process indicators in the context of comparative evaluation of the performance of healthcare providers and professionals. Cardiac care has a relevant role in this evaluation process and is performed through crude and risk-adjusted indicators among AMI patients [18].

Our findings suggest the importance of including travel time as a confounding factor in comparative outcome analysis based on hospital quality indicators. We consider that such utilization is appropriate since travel time is a reliable proxy of pre-hospital system delay and a strong predictor of 30-day mortality after timely execution of PCI for STEMI.

## Conclusion

We measured the effect of travel time delay on STEMI 30-day mortality after PCI. Our results indicate that travel time, even among patients treated timely with reperfusion, has a strong effect on mortality. Efforts of health care systems should be focused on the reduction of both pre-hospital and in-hospital delay. Travel time can be used to better estimate patient’s mortality risk in comparative evaluation of hospital quality of care.

## Supporting Information

S1 Fig Ratios between average speeds according to origin/destination of route and time bands and average regional value. (DOC)

S1 File Details on methodology to accounting for traffic effect. (DOC)

S1 Table List of conditions and relative ICD-9-CM codes for identification of comorbidities. (DOC)

S2 Table Average speed, length of route, and travel time according to time bands after application of the effect of traffic in the area of the municipality of Rome. Metropolitan = Inside municipality of Rome; Measures are stratified according to the three time bands defined during the daytime and refers to the route used for calculation of travel times. Routes origin or destination were classified as follows: metropolitan, if lying in the municipality of Rome, countryside, if lying out of the municipality of Rome. Average speed was calculated on the basis of travel time after the application of the correction factor for traffic during the hours from 8,00 to 21,59.
(DOC)

S3 Table Logistic regression analysis for <=120 minutes door to balloon cohort stratified by time band. EMS, Emergency Medical Service;* Index admission.
(DOC)
