# Supplementary material for: The Tradeoff between Travel Time from Home to Hospital and Door to Balloon Time in Determining Mortality among STEMI Patients Undergoing PCI
Source: PLoS One. 2016 Jun 23;11(6):e0158336. doi: 10.1371/journal.pone.0158336 (PMC4918978; doi:10.1371/journal.pone.0158336)
Supplement: S1 Fig — (DOC) [file pone.0158336.s001.doc]

**S1 Fig:** Ratios between average speeds according to origin/destination of route and time bands and average regional value
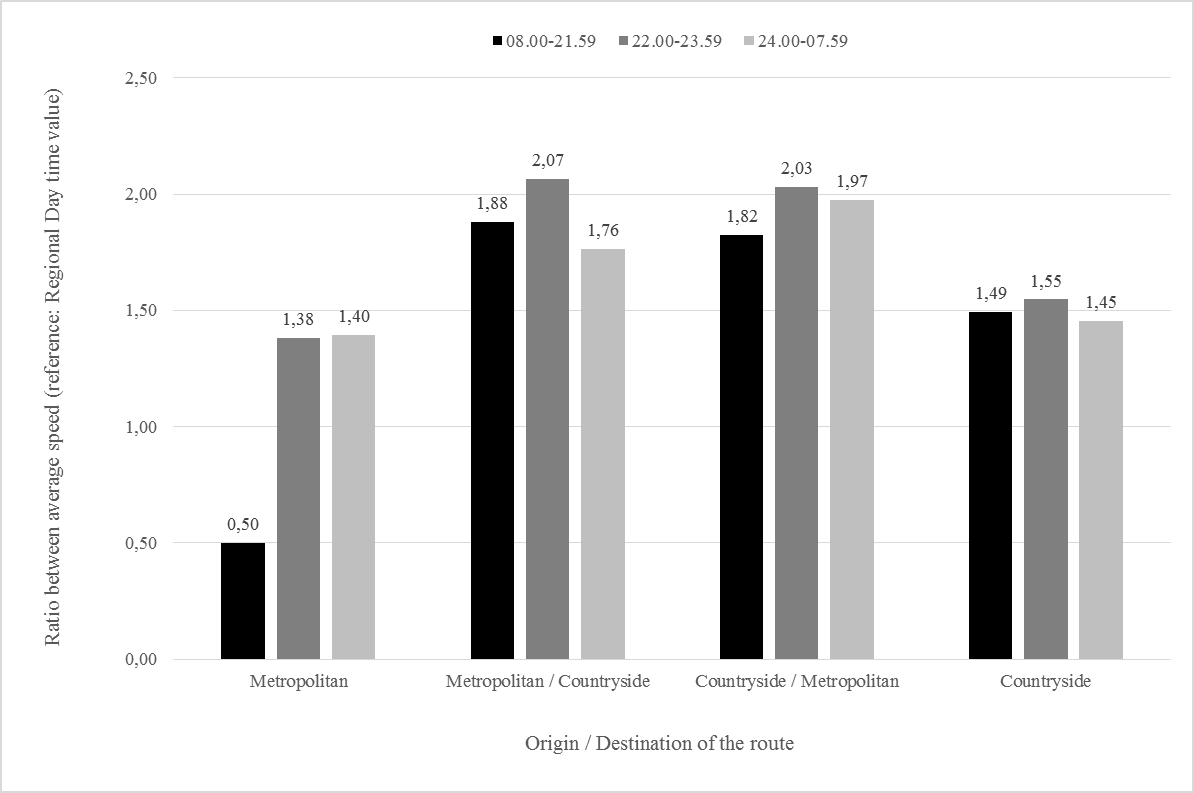
.
