# Supplementary material for: The Tradeoff between Travel Time from Home to Hospital and Door to Balloon Time in Determining Mortality among STEMI Patients Undergoing PCI
Source: PLoS One. 2016 Jun 23;11(6):e0158336. doi: 10.1371/journal.pone.0158336 (PMC4918978; doi:10.1371/journal.pone.0158336)
Supplement: S1 File — (DOC) [file pone.0158336.s002.doc]

**S1 File: Details on methodology to accounting for traffic effect.**

We applied a method to take into account the effect of traffic in the municipality of Rome and to estimate a measure of travel time adjusted for traffic (TTAT). The effect of traffic in Rome was estimated among several time bands (8.00-21.59, 22.00-23.59 and 0-7.59 AM) which were assumed as optimal to explain differences in traffic during daytime in the city. For a sample of routes, a traffic correction factor (TC) was estimated and applied to network route analysis data provided by ESRI. The statistical units were the routes from each study member address of residence to each hospital.

The following information was considered:

A. The minimum driving travel time (MTT), estimated by ESRI

B. The ground way public transport travel times (PT), derived from ATAC (Urban mobility operator for the city of Rome)

C. The number of cars that runs along a route for each time band, estimated by the Rome municipality Environment protection department - Civil protection unit

The traffic correction factor was obtained following several steps:

A. The mean number of cars, for each time band, was weighted for the length of routes that was run (WMC – weighted medium number of cars)

B. The difference between ESRI minimum driving travel time (MTT) and ATAC public transport travel time (PT) was calculated obtaining a measure that was called DBPT

C. The traffic coefficient (TC) was estimated from a linear regression model between DBPT and WMC

D. The travel time due to traffic (TTT) was obtained by multiplying WMC for the estimated traffic coefficient (TC). This value was defined for the full day and for time bands

E. Travel time due to traffic (TTT) was added to the minimum driving travel time (MTT) to estimate travel time adjusted for traffic (TTAT)

We applied the correction for traffic effects to travel times of patients experiencing a STEMI during the 8.00-21.59 time band with associated routes lying in the area of the municipality of Rome.
