# Supplementary material for: The Tradeoff between Travel Time from Home to Hospital and Door to Balloon Time in Determining Mortality among STEMI Patients Undergoing PCI
Source: PLoS One. 2016 Jun 23;11(6):e0158336. doi: 10.1371/journal.pone.0158336 (PMC4918978; doi:10.1371/journal.pone.0158336)
Supplement: S1 Table — (DOC) [file pone.0158336.s003.doc]

**S1 Table: List of conditions and relative ICD-9-CM codes for identification of comorbidities.**

| Condition | ICD-9-CM code | |
| --- | --- | --- |
|  | During the episode of AMI / during index ED access | During previous hospitalizations or Emergency Department accesses |
| Cancer | 140.0–208.9 | 140.0–208.9 |
| Diabetes | 250.0-250.9 | 250.0-250.9 |
| Lipid metabolism disturbances | 272 | 272 |
| Obesity | 278.0 | 278.0 |
| Blood disorders | 280-285, 288, 289 | 280-285, 288, 289 |
| Hypertension | 401-405 | 401-405 |
| Previous myocardial infarction | 412 | 410, 412 |
| Other forms of ischemic heart disease |  | 411, 413, 414 |
| Heart failure |  | 428 |
| Ill-defined descriptions or complications of heart disease |  | 429 |
| Rheumatic heart disease | 393-398 | 391, 393-398 |
| Cardiomyopathy | 425 | 425 |
| Acute endocarditis and myocarditis |  | 421, 422 |
| Other heart conditions | 745, V15.1, V42.2, V43.2, V43.3, V45.0 | 745, V15.1, V42.2, V43.2, V43.3, V45.0 |
| Conduction disturbances and arrhythmias |  | 426, 427 |
| Cerebrovascular disease | 433, 437, 438 | 430-432, 433, 434, 436, 437, 438 |
| Vascular disease | 440-448 (excluding 441.1, 441.3, 441.5, 441.6, 444) | 440-448, 557 |
| Chronic obstructive pulmonary disease (COPD) | 491-492, 494, 496 | 491-492, 494, 496 |
| Chronic renal disease | 582-583, 585-588 | 582-583, 585-588 |
| Chronic diseases (liver, pancreas, intestine) | 571-572, 577.1-577.9, 555, 556 | 571-572, 577.1-577.9, 555, 556 |
| Previous coronary artery bypass graft | V45.81 | 36.1, V45.81 |
| Previous coronary angioplasty | V45.82 | 36.0, V45.82 |
| Cerebral revascularization procedures |  | 38.01, 38.02, 38.11, 38.12, 38.31, 38.32 |
| Other cardiac operations |  | 35, 37.0, 37.1, 37.3, 37.4, 37.5, 37.6, 37.9 |
| Other vascular operations |  | 38-39.5, excluding: 38.01, 38.02, 38.5, 38.11, 38.12, 38.31, 38.32, 38.93 |
