# Supplementary material for: The Tradeoff between Travel Time from Home to Hospital and Door to Balloon Time in Determining Mortality among STEMI Patients Undergoing PCI
Source: PLoS One. 2016 Jun 23;11(6):e0158336. doi: 10.1371/journal.pone.0158336 (PMC4918978; doi:10.1371/journal.pone.0158336)
Supplement: S2 Table — Metropolitan = Inside municipality of Rome; Measures are stratified according to the three time bands defined during the daytime and refers to the route used for calculation of travel times. Routes origin or destination were classified as follows: metropolitan, if lying in the municipality of Rome, countryside, if lying out of the municipality of Rome. Average speed was calculated on the basis of travel time after the application of the correction factor for traffic during the hours from 8,00 to 21,59. (DOC) [file pone.0158336.s004.doc]

**S2 Table:** Average speed, length of route, and travel time according to time bands after application of the effect of traffic in the area of the municipality of Rome.

| **Origin** | **Destination** |  | **Hours interval** | | | | | | | | | | | | | |  | **All** | | | |
| --- | --- | --- | --- | --- | --- | --- | --- | --- | --- | --- | --- | --- | --- | --- | --- | --- | --- | --- | --- | --- | --- |
|  |  |  | **08.00-21.59** | | | |  | **22.00-23.59** | | | |  | **24.00-07.59** | | | |  |  |  |  |  |
|  |  |  | **N** | **Mean** | | |  | **N** | **Mean** | | |  | **N** | **Mean** | | |  | **N** | **Mean** | | |
|  |  |  |  | **Speed (Km/h)** | **Length (Km)** | **Travel time (Minutes)** |  |  | **Speed (Km/h)** | **Length (Km)** | **Travel time (Minutes)** |  |  | **Speed (Km/h)** | **Length (Km)** | **Travel time (Minutes)** |  |  | **Speed (Km/h)** | **Length (Km)** | **Travel time (Minutes)** |
|  |  |  |  |  |  |  |  |  |  |  |  |  |  |  |  |  |  |  |  |  |  |
| **Metropolitan** | **Metropolitan** |  | 1,738 | 19.2 | 6.5 | 19.2 |  | 143 | 53.0 | 6.0 | 6.4 |  | 443 | 53.6 | 6.3 | 6.5 |  | 2,324 | 27.8 | 6.4 | 16.0 |
|  | **Countryside** |  | 41 | 72.2 | 49.6 | 37.3 |  | 5 | 79.3 | 48.5 | 35.0 |  | 8 | 67.7 | 29.4 | 21.5 |  | 54 | 72.2 | 46.5 | 34.8 |
| **Countryside** | **Metropolitan** |  | 248 | 70.0 | 37.8 | 30.7 |  | 20 | 77.9 | 31.5 | 24.2 |  | 60 | 75.8 | 37.8 | 28.1 |  | 328 | 71.6 | 37.4 | 29.9 |
|  | **Countryside** |  | 698 | 57.3 | 15.9 | 14.8 |  | 47 | 59.4 | 13.9 | 13.1 |  | 157 | 55.8 | 12.9 | 12.9 |  | 902 | 57.1 | 15.2 | 14.3 |
| **All** | |  | 2,725 | 32.4 | 12.1 | 19.8 |  | 215 | 57.1 | 10.8 | 9.8 |  | 668 | 56.4 | 10.8 | 10.0 |  | 3,608 | 38.4 | 11.8 | 17.3 |

Metropolitan= Inside municipality of Rome

**S1 Graph:** Ratios between average speeds according to origin/destination of route and time bands and average regional value
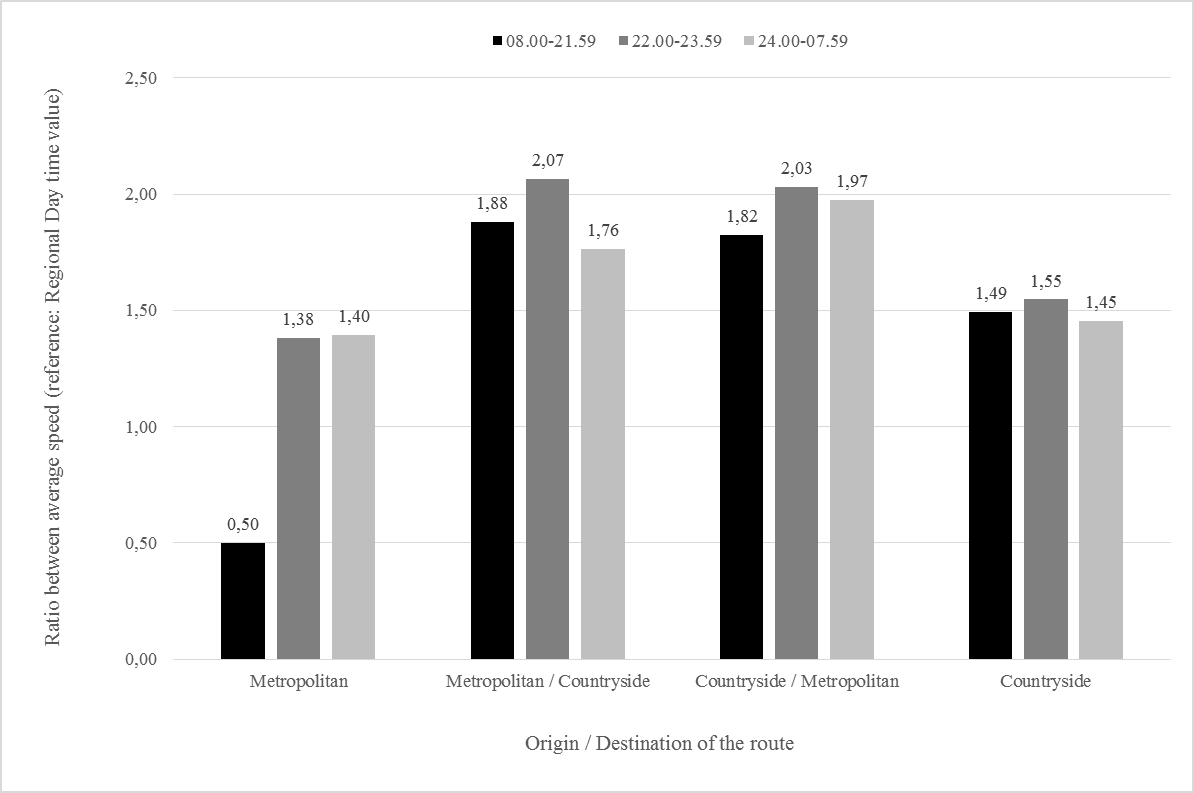
.

**S3 Table: Logistic regression analysis for <=120 minutes door to balloon cohort stratified by time band.**

|  |  |  | **All (N=3.327)** | | | |  | **Time band** | | | | | | | | |
| --- | --- | --- | --- | --- | --- | --- | --- | --- | --- | --- | --- | --- | --- | --- | --- | --- |
|  |  |  |  |  |  |  |  | **22.00-7.59 (N=822)** | | | |  | **08.00-21.59 (N=2.505)** | | | |
| **Parameter** | **Category** |  | **Odds ratio estimate** | **95% Confidence limits** | | **P** |  | **Odds ratio estimate** | **95% Confidence limits** | | **P** |  | **Odds ratio estimate** | **95% Confidence limits** | | **P** |
|  |  |  |  |  |  |  |  |  |  |  |  |  |  |  |  |  |
|  |  |  |  |  |  |  |  |  |  |  |  |  |  |  |  |  |
| Travel time | > 14 minutes |  | 1.46 | 0.94 | 2.25 | 0.09 |  | 2.65 | 0.90 | 7.80 | 0.08 |  | 1.37 | 0.82 | 2.28 | 0.23 |
| Gender | Male |  | 0.71 | 0.45 | 1.13 | 0.15 |  | 0.61 | 0.23 | 1.60 | 0.31 |  | 0.76 | 0.45 | 1.28 | 0.30 |
| Age class | 65-84 years vs 35-64 years |  | 3.19 | 1.83 | 5.57 | <.0001 |  | 6.27 | 1.70 | 23.11 | 0.01 |  | 2.68 | 1.44 | 5.01 | 0.00 |
|  | > 84 years vs 35-64 years |  | 9.88 | 4.74 | 20.58 | <.0001 |  | 16.88 | 2.67 | 53.11 | 0.00 |  | 9.04 | 4.00 | 20.43 | <.0001 |
| Systolic blood pressure | ≤100 mmHg |  | 3.57 | 2.23 | 5.73 | <.0001 |  | 2.87 | 1.04 | 7.91 | 0.04 |  | 3.71 | 2.17 | 6.36 | <.0001 |
| Presentation | E.M.S vs. Direct |  | 1.51 | 0.93 | 2.44 | 0.10 |  | 2.78 | 0.89 | 8.67 | 0.08 |  | 1.27 | 0.74 | 2.19 | 0.38 |
| Comorbidities | Other heart conditions |  | 5.11 | 1.55 | 16.88 | 0.01 |  | 1.79 | 0.12 | 25.69 | 0.67 |  | 6.46 | 1.61 | 25.86 | 0.01 |
|  | Diabetes* |  | 1.99 | 1.23 | 3.23 | 0.01 |  | 2.87 | 1.10 | 7.47 | 0.03 |  | 1.76 | 0.99 | 3.12 | 0.05 |
|  | Cerebrovascular diseases |  | 2.75 | 1.33 | 5.70 | 0.01 |  | 1.70 | 0.18 | 16.14 | 0.65 |  | 3.15 | 1.43 | 6.92 | 0.00 |
|  | Cancer |  | 2.11 | 0.96 | 4.60 | 0.06 |  | 2.29 | 0.44 | 12.07 | 0.33 |  | 2.15 | 0.88 | 5.29 | 0.09 |

EMS, Emergency Medical Service;

* Index admission
