# Supplementary material for: The Tradeoff between Travel Time from Home to Hospital and Door to Balloon Time in Determining Mortality among STEMI Patients Undergoing PCI
Source: PLoS One. 2016 Jun 23;11(6):e0158336. doi: 10.1371/journal.pone.0158336 (PMC4918978; doi:10.1371/journal.pone.0158336)
Supplement: S3 Table — EMS, Emergency Medical Service;* Index admission. (DOC) [file pone.0158336.s005.doc]

**S3 Table: Logistic regression analysis for <=120 minutes door to balloon cohort stratified by time band.**

|  |  |  | **All (N=3.327)** | | | |  | **Time band** | | | | | | | | |
| --- | --- | --- | --- | --- | --- | --- | --- | --- | --- | --- | --- | --- | --- | --- | --- | --- |
|  |  |  |  |  |  |  |  | **22.00-7.59 (N=822)** | | | |  | **08.00-21.59 (N=2.505)** | | | |
| **Parameter** | **Category** |  | **Odds ratio estimate** | **95% Confidence limits** | | **P** |  | **Odds ratio estimate** | **95% Confidence limits** | | **P** |  | **Odds ratio estimate** | **95% Confidence limits** | | **P** |
|  |  |  |  |  |  |  |  |  |  |  |  |  |  |  |  |  |
|  |  |  |  |  |  |  |  |  |  |  |  |  |  |  |  |  |
| Travel time | > 14 minutes |  | 1.46 | 0.94 | 2.25 | 0.09 |  | 2.65 | 0.90 | 7.80 | 0.08 |  | 1.37 | 0.82 | 2.28 | 0.23 |
| Gender | Male |  | 0.71 | 0.45 | 1.13 | 0.15 |  | 0.61 | 0.23 | 1.60 | 0.31 |  | 0.76 | 0.45 | 1.28 | 0.30 |
| Age class | 65-84 years vs 35-64 years |  | 3.19 | 1.83 | 5.57 | <.0001 |  | 6.27 | 1.70 | 23.11 | 0.01 |  | 2.68 | 1.44 | 5.01 | 0.00 |
|  | > 84 years vs 35-64 years |  | 9.88 | 4.74 | 20.58 | <.0001 |  | 16.88 | 2.67 | 53.11 | 0.00 |  | 9.04 | 4.00 | 20.43 | <.0001 |
| Systolic blood pressure | ≤100 mmHg |  | 3.57 | 2.23 | 5.73 | <.0001 |  | 2.87 | 1.04 | 7.91 | 0.04 |  | 3.71 | 2.17 | 6.36 | <.0001 |
| Presentation | E.M.S vs. Direct |  | 1.51 | 0.93 | 2.44 | 0.10 |  | 2.78 | 0.89 | 8.67 | 0.08 |  | 1.27 | 0.74 | 2.19 | 0.38 |
| Comorbidities | Other heart conditions |  | 5.11 | 1.55 | 16.88 | 0.01 |  | 1.79 | 0.12 | 25.69 | 0.67 |  | 6.46 | 1.61 | 25.86 | 0.01 |
|  | Diabetes* |  | 1.99 | 1.23 | 3.23 | 0.01 |  | 2.87 | 1.10 | 7.47 | 0.03 |  | 1.76 | 0.99 | 3.12 | 0.05 |
|  | Cerebrovascular diseases |  | 2.75 | 1.33 | 5.70 | 0.01 |  | 1.70 | 0.18 | 16.14 | 0.65 |  | 3.15 | 1.43 | 6.92 | 0.00 |
|  | Cancer |  | 2.11 | 0.96 | 4.60 | 0.06 |  | 2.29 | 0.44 | 12.07 | 0.33 |  | 2.15 | 0.88 | 5.29 | 0.09 |

EMS, Emergency Medical Service;

* Index admission
